# Risk factors for recurrent intussusception after successful reduction in pediatric patients in a tertiary care hospital of Nepal: A prospective study

**DOI:** 10.1016/j.amsu.2022.103427

**Published:** 2022-04-04

**Authors:** Shankar Adhikari, Dinesh Prasad Koirala, Rameshwor Prasad Pokhrel, Geha Raj Dahal, Sanjeev Kharel, Subita Neupane

**Affiliations:** a Department of GI and General Surgery, Tribhuvan University Teaching Hospital, Institute of Medicine, Kathmandu, Nepal; b Department of GI and General Surgery, Pediatric Surgery Unit, Tribhuvan University Teaching Hospital, Institute of Medicine, Kathmandu, Nepal; c Maharajgunj Medical Campus, Tribhuvan University, Institute of Medicine, Kathmandu, Nepal; d Department of General Practice and Emergency Medicine, Bir Hospital, Kathmandu, Nepal

**Keywords:** *Intussusception*, *Hydrostatic reduction*, *Recurrence*, *Risk factors*

## Abstract

**Background:**

Intussusception is defined as the invagination of one segment of intestine into another segment of intestine. It may recur because of persistence or return of some factor responsible for the primary intussusception. Various risk factors have been reported but still not well elucidated.

**Materials and methods:**

This is the prospective observational study. In this study, 78 patients, age <16 years with diagnosis of intussusception between June 2019 and April 2020 who had successful reduction with either hydrostatic reduction and/or operative reduction in Teaching Hospital were enrolled in the study. This is study of early recurrence as patients were followed up to a period of 1 month for recurrence of intussusception. The recurrent cases were thus identified and various variables were compared between recurrent and non-recurrent cases by univariable and multivariable analysis.

**Results:**

Among 78 patients, 13 patients (16.7%) had recurrent intussusception. In the univariable analysis model, the significant risk factors for recurrence of intussusception analyzed were duration of symptoms of 48 h or more, fever, blood in stool and palpable mass. While after multivariable analysis, we found that the significant risk factors for recurrence of intussusception were duration of symptoms ≥48 h (OR = 5.32, p-value = 0.047), Fever (OR = 17.32, p-value = 0.001), palpable mass (OR = 24.12, p-value = 0.017).

**Conclusion:**

Attention and awareness among pediatricians about these sonographic and clinical risk factors especially symptoms for recurrence are needed to minimize pre-hospital delay and identify patients in risk of recurrence. This ultimately helps to improve care for pediatric patients with recurrent intussusception.

## Highlights

• Intussusception is a common abdominal emergency in infancy and childhood.
• Definitive or reliable risk factors for recurrence, other than anatomical features, have not been well elucidated.
• Duration of symptoms >24 h, fever and palpable mass are the risk factors.

## 1 Background

Intussusception is a common abdominal emergency in infancy and childhood with an incidence of 1–4 in 2000. It is defined as the invagination of one segment of intestine into another segment of intestine [1]. The classical picture of intussusception is vomiting, currant jelly stools and a palpable abdominal mass. But, less than 25% of children presents with above symptoms, resulting in delay in diagnosis. Delayed diagnosis and treatment may lead to bowel necrosis or even death [2]. Intussusception can be either idiopathic or secondary to pathological lead point including Meckel's diverticulum, duplication, polyps and tumors. Most of the cases (90%) are idiopathic which means there is no obvious cause other than lymphoid hyperplasia of terminal ileum. Clark and Bunts reported the first case of recurrence of intussuception in 7-month old child which was managed expectantly resulting in ultimate death of the child [3]. It is evident that intussusception may recur because of persistence or return of some factor responsible for the primary intussusception.

The treatment for intussusception is both nonoperative and operative. Non-operative reduction is usually the primary treatment and can be performed with hydrostatic or pneumatic pressure enema under ultrasound or fluoroscopy. While, operative reduction is reserved for those cases with failed nonoperative reduction or cases with peritonitis. The recurrence rate for non-operative reduction is reported to be up to 20% [4,5]. While, the recurrence following operative reduction is about 1%–3% [6]. The post-operative adhesions play a vital role in preventing recurrence. A recent structured literature review and meta-analysis found an overall recurrence rate of 12.7% [7].

To date, definitive or reliable risk factors for recurrence, other than anatomical features, have not been well elucidated. Awareness of the recurrence of intussusceptions is lacking and early diagnosis, is often challenging for pediatricians in clinical setting. Accordingly, the aim of this study was to identify clinical features and risk factors for recurrent intussusception, beyond the anatomical points previously identified, which would allow clinicians to identify patients who are at an increased risk for recurrence after a primary episode. Mainly the sonographic and clinical risk factors for recurrence were given emphasized. Providing a clinical index for the risk of intussusception recurrence would allow pediatricians to plan a more appropriate course of management and follow-up for these patients.

## 2 Materials and methods

This prospective observational study was approved by the ethics committees of Institutional review board of Tribhuvan University, Institute of Medicine. Between June 2019 and April 2020, all pediatric patients with intussusception treated with nonoperative and operative reduction in Teaching Hospital were followed up for recurrence and data were collected. This is the study of early recurrence with only one month follow-up. Recurrence of intussusception (RI) was defined as intussusception that recurred after the first successful reduction with nonoperative and/or operative reduction. The time of follow-up was one month. This paper was registered in Clinical Trials.gov with identification number NTC05259670. This study is reported according to STROCSS criteria [8].

### 2.1 Inclusion and exclusion criteria

We included the patients who were diagnosed with intussusception from the age of 0 year–16 years who received nonoperative and operative reduction as an initial treatment. Pediatric patients with spontaneous reduction of hydro-reduction and who required resection and anastomosis as a part of operative procedure were excluded from the study.

### 2.2 Details on procedures done

In all cases the final diagnosis of intussusception was made by clinical features together with abdominal ultrasonography. Once the diagnosis was made, informed consent was obtained from the child's parents or guardians, before proceeding with the reduction procedure.

In child with no features of peritonitis or cardiovascular compromise, the reduction was performed using a water enema under ultrasound guidance. For this, the child was placed in supine position and a 10–12 Fr Foley's catheter was inserted into child's rectum. Foley's catheter was connected to 1 L of lukewarm normal saline suspended at a height of about 100–120 cm above procedure table. The thighs were pressed together manually to ensure a tight anal seal and fluid was infused by gravity into bowel. The flow of enema fluid into colon and retrograde motion of intussusception towards ileocaecal valve was visualized with abdominal sonography. The reduction procedure consisted of one to three attempts, each for a maximum of 3 min and colon was drained if complete reduction was not achieved by maintaining hydrostatic pressure over 3 min. Successful hydro reduction was ensured by disappearance of target sign, visualization of fluid reflux into distal ileum from caecum, fluid filling of small bowel loops. Post procedure, the child was kept under medical supervision with nil per os. Ultrasonography was repeated in all cases after 12 h of reduction and child was allowed to feed if there was no recurrence of intussusception and discharged from hospital. In cases in which a reduction by saline enema failed of if the child showed any signs of perforation or cardiovascular compromise, we proceeded with emergent surgery. Postoperatively, the child was managed and discharged according to standard protocols depending upon the intervention done.

Child was followed by telephonic calls on end of first week and on end of month after reduction to inquire about any symptoms of intussusception. Repeat ultrasonography was done wherever deemed necessary. Recurrent intussusception was defined as recurrence of invagination after the initial successful reduction, regardless of the procedure used (hydro-reduction or surgery).

**Fig. 1:**
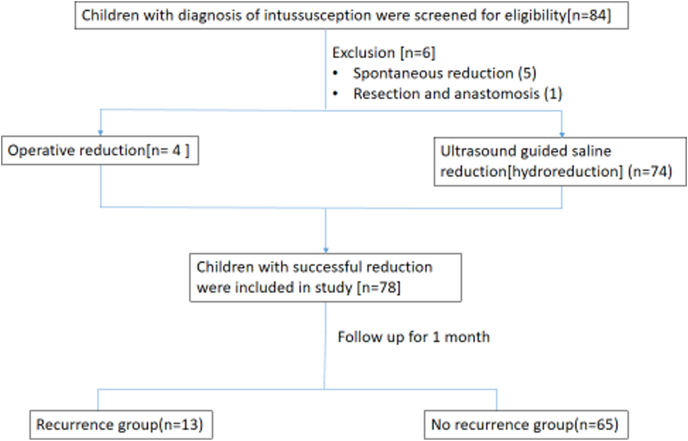
Study flow of patients diagnosed with intussusception.

The study flow of our study is depicted in Fig. 1. The demographic features, clinical findings, ultrasonography findings and laboratory values at baseline were compared between the non-recurrence and recurrence group to find the sonographic and clinical risk factors by univariate and multivariate analysis.

### 2.3 Variables

A standard structured questionnaire was filled by interviewing the patient (if possible) and family members/relatives after taking informed written consent. The questionnaire documented the patient's age, sex, weight, duration of symptoms, presenting symptoms: abdominal pain, excessive cry, vomiting, lethargy, blood in stool, fever, palpable mass, constipation, diarrhoea, location of mass, enlarged lymph nodes (LNs), blood counts and methods of reduction. Age of 2 years, weight of 12 kg and duration of symptoms of 48 h was used to classify patients into two groups. Data on recurrent and non-recurrent cases of intussusception were compared as per above headings.

### 2.4 Statistical analysis

Statistical analysis was performed using SPSS version 23.0. The descriptive data were reported in count and percent for categorical data, and mean and standard deviation or median and interquartile range for continuous data. Differences were evaluated using Student's *t*-test for continuous parametric data, the Wilcoxon test for continuous nonparametric data and Pearson's chi-squared test for noncontinuous data. Logistic regression was performed to identify independent risk factors. A p value < 0.05 was considered statistically significant.

## 3 Results

### 3.1 Patient selection

Eighty-four patients were screened for eligibility to enroll in study after diagnosis of intussusception. Six patients were excluded from study due to spontaneous reduction and resection anastomosis. No one was lost to follow-up. Seventy-eight patients were included in study out of which 74 underwent ultrasound guided saline reduction and 4 underwent operative reduction following failed attempts of hydro-reduction.

### 3.2 Patient characteristics

Among the 78 total patients in our study, 55 were male (71%) and 23 were female (29%), with a median age of 20.72 ± 16.9 months (range 5–110 months). The male to female ratio was 2:1. The median weight of patient was 10 kg (range 8–13 kg). The median interval time from symptom onset to presentation was approximately 28 h. The most common symptoms were abdominal pain, excessive cry and vomiting (97%, 95% and 54% respectively). The most common location of the mass as identified by sonography was on right side of abdomen and this was found in 97% of the patients. The characteristics of the study population are described in Table 1.

**Table 1:** Baseline characteristics of the patients (n = 78).

Characteristics	n (%), Median (25, 75)^a^
**Age (months)**	17 (9, 26)
**Age (years)**	
< 2 years	53 (67.95)
≥ 2 years	25 (32.05)
**Sex**	
Male	55 (70.51)
Female	23 (29.49)
**Weight (kg)**	10 (8, 13)
**Weight category (kg)**	
<12 kg	52 (66.67)
≥12 kg	26 (33.33)
**Duration of symptoms (Hours)**	27.5 (20, 47)
**Duration of symptoms (Hours**)	
< 48 h	59 (75.64)
≥ 48 h	19 (24.36)
**Abdominal pain**	
No	2 (2.56)
Yes	76 (97.44)
**Excessive cry**	
No	4 (5.13)
Yes	74 (94.87)
**Vomiting**	
No	36 (46.15)
Yes	42 (53.85)
**Lethargy**	
No	53 (67.95)
Yes	25 (32.05)
**Blood in stool**	
No	56 (71.79)
Yes	22 (28.21)
**Fever**	
No	59 (75.64)
Yes	19 (24.36)
**Palpable mass**	
No	73 (93.59)
Yes	5 (6.41)
**Constipation**	
No	76 (97.44)
Yes	2 (2.56)
**Diarrhoea**	
No	77 (98.72)
Yes	1 (1.28)
**Maximum dimension (cm)**^b^	2.97 ± 0.62
**Location**	
Subhepatic	75 (96.15)
Left iliac fossa	3 (3.85)
**Enlarged lymph nodes (> 1 cm)**	
No	39 (50)
Yes	39 (50)
**Leukocyte (/mm3)**^**b**^	10949.49 ± 4090.53
**Neutrophil (%)**^**b**^	54.60 ± 14.37
**Lymphocyte (%)**^**b**^	37.08 ± 14.87
**Method of reduction**	
• Hydro-reduction	
• Operation	

a Denotes median, and interquartile range for non-normally distributed data.

b Denotes mean and standard deviation for normally distributed data.

### 3.3 Comparison between the non-recurrence group and recurrence group

Recurrent intussusception was identified in 13 patients (16.7%). Most of the recurrences (56%) were seen within 6 h following saline reduction while no recurrence was seen in patients who underwent operative reduction during one month follow up.

The risk factors for recurrence of intussusception were analyzed by univariable analysis (Table 2) and multivariable analysis (Table 3). In the univariable analysis model, the significant risk factors for recurrence of intussusception analyzed were duration of symptoms (≥48 h), fever, blood in stool and palpable mass (p-value for each parameter are stated in Table 2). When the duration of symptoms was dichotomized (<48 h and ≥48 h), the rate of recurrence was higher for patients presenting ≥48 h (54%,7/13 patients) compared to patients presenting <48 h (18%,12/65 patients; p-value = 0.0067). After multivariable analysis was done, we found that the significant risk factors for recurrence of intussusception were duration of symptoms ≥48 h (OR = 5.32, p-value = 0.047), Fever (OR = 17.32, p-value = 0.001), palpable mass (OR = 24.12, p-value = 0.017) as shown in Table 3.

**Table 2:** Univariate comparison of recurrence versus no recurrence group (n = 78).

Characteristics	Non-recurrence (n = 65) n (%)	Recurrence (n = 13) n (%)	P-value [1,2]
**Recurrence, % (95% CI)**	83.3 (73.1–90.2)	16.7 (9.8–26.9)	
**Age (years)**			
< 2 years	45 (69.23)	8 (61.54)	0.587
≥ 2 years	20 (30.77)	5 (38.46)	
**Sex**			
Male	47 (72.31)	8 (61.54)	0.437
Female	18 (27.69)	5 (38.46)	
**Weight category (kg)**			
<12 kg	44 (67.69)	8 (61.54)	0.667
≥12 kg	21 (32.31)	5 (38.46)	
**Duration of symptoms (Hours**)			
< 48 h	53 (81.54)	6 (46.15)	0.007*
≥ 48 h	12 (18.46)	7 (53.85)	
**Abdominal pain**			
No	1 (1.54)	1 (7.69)	0.307
Yes	64 (98.46)	12 (92.31)	
**Excessive cry**			
No	3 (4.62)	1 (7.69)	0.525
Yes	62 (95.38)	12 (92.31)	
**Vomiting**			
No	28 (43.08)	8 (61.54)	0.223
Yes	37 (56.92)	5 (38.46)	
**Lethargy**			
No	45 (69.23)	8 (61.54)	0.587
Yes	20 (30.77)	5 (38.46)	
**Blood in stool**			
No	50 (76.92)	6 (46.15)	0.024*
Yes	15 (23.08)	7 (53.85)	
**Fever**			
No	56 (86.15)	3 (23.08)	<0.001*
Yes	9 (13.85)	10 (76.92)	
**Palpable mass**			
No	63 (96.92)	10 (76.92)	0.007*
Yes	2 (3.08)	3 (23.08)	
**Constipation**			
No	64 (98.46)	12 (92.31)	0.307
Yes	1 (1.54)	1 (7.69)	
**Diarrhoea**			
No	64 (98.46)	13 (100)	1.000
Yes	1 (1.54)	0	
**Location**			
Subhepatic	64 (98.46)	11 (84.62)	0.070
Left iliac fossa	1 (1.54)	2 (15.38)	
**Enlarged lymph nodes (> 1 cm)**			
No	34 (52.31)	5 (38.46)	0.362
Yes	31 (47.69)	8 (61.54)	
**Leukocyte (/mm3), mean** ± **SD**^a^	11262.15 ± 4321.97	9386.15 ± 2136.69	0.132
**Neutrophil (%), mean** ± **SD**^a^	54.56 ± 15.06	54.76 ± 10.72	0.963
**Lymphocyte (%), mean** ± **SD**^a^	37.24 ± 15.73	36.30 ± 9.94	0.837

[1,2] denotes for chi-square test used for ≥5 value in each contingency table, and fisher exact test for <5 value in contingency table.

*denotes for statistically significant at p < 0.05.

a Denotes for independent *t*-test for continuous variables.

**Table 3:** Multivariate analysis of risk factors with recurrence versus non-recurrence group (n = 78).

Characteristics	Bivariate analysis COR (95% CI)	P-value^a^	Multivariate analysis AOR (95% CI)	P-value^b^
**Duration of symptoms (Hours**)				
< 48 h	Ref	0.011*	Ref	0.047*
≥ 48 h	5.12 (1.46–18.12)		5.32 (0.95–29.71)	
**Blood in stool**				
No	Ref	0.031*	Ref	0.138
Yes	3.88 (1.13–13.35)		3.68 (0.65–20.58)	
**Fever**				
No	Ref	<0.001*	Ref	0.001*
Yes	20.74 (4.77–90.18)		17.32 (2.98–100.3)	
**Palpable mass**				
No	Ref	0.021*	Ref	0.017*
Yes	9.45 (1.40–63.78)		24.12 (1.76–330)	

COR: Crude odds ratio AOR: Adjusted odds ratio.

* denotes statistically significant at p < 0.05.

a Denotes binary logistic regression analysis for non-adjusted model.

b Denotes for multivariate binary logistic regression for adjusted model including duration of symptoms (hours), blood in stool, fever, and palpable mass.

## 4 Discussion

Recurrent intussusception was first reported by Clark and Bunts in 1900 and since then, several cases have been reported with rates as high as 20% [3,4]. Although the recurrence of intussusception is considered to be spontaneous [9], specific predisposing factors have been identified, including polyps and Meckel's diverticulum, both of which are associated with relatively high recurrence rate [10]. Still, little is known about the factors mainly sonographic and clinical risk factors associated with recurrence of intussusception in infants and children and reports have been widely inconsistent.

Lee et al. in their retrospective review of 137 patients has found 23 recurrences (recurrence rate; 16.8%) [11]. This recurrence rate is consistent with recurrence rate observed in our study (16.7%). Some of the reviewed literature mentioned about the patient's age as a risk factor for recurrence of intussusception. In their review of 42 cases of recurrent intussusception, Xie et al. showed that 66.67% of the recurrent intussusception were found in pediatric patients older than 2 years [1]. Lee et al. reported a rate of recurrence of 95.7% among patients over the age of 1 year at the time of first diagnosis [11]. However, we could not find any significant differences in age of patients between recurrent and non-recurrent group with only 39% of patient aged 2 years or older in recurrent group. Our study is the study of early recurrence. Different studies have shown prevalence of early recurrence. Guo et al. found early recurrence rate (within 24 h) 6.5% [12]. While, Gilmore et al. in 15-year period among 117 pediatric patients showed early recurrence rate 5.3% only while McDermott et al. found 7% recurrence rate [13,14].

The clinical symptoms related to intussusception recurrence have previously been studied. Xie et al. identified duration of symptoms ≥48 h, rectal bleeding, location of mass (left over right) as risk factors of recurrence [1]. Similarly, a recent systematic review and meta-analysis showed children with fever and pathological lead point had higher recurrence risk following enema reduction. While, prevalence of vomiting was found to be low in recurrent cases in comparison to non-recurrent cases [15]. Our study also showed that recurrence of intussusception is higher in patients presenting late and in those with fever, blood in stool and mass over left side of abdomen but no significant findings on vomiting was seen.

Intussusception caused by pathological lead points might more easily lead to recurrence. In our study, among recurrent cases, we had only one case with obvious pathological cause i.e., Meckel's diverticulum which was identified during operative intervention. Considering that the evaluations including Computed Tomography, endoscopy, scintigraphy or exploratory surgery were not routinely conducted, conclusions about the association between pathological lead points and recurrence of intussusception could not been established. Furthermore, Niramis et al. found that pathological lead points were noted only in 7 (9.3%) of the 75 patients with recurrent intussusception [16]. Some recurrences are inexplicable except by the oft-repeated phrase that repeat intussusception are due to the same combination of factors which precipitated primary intussusception.

In our study, enlarged LNs were observed on Ultrasound of abdomen in about half (50%) of our patients (n = 39). Although, enlarged lymph nodes were present in 61% of patients with recurrence, we did not identify any significant difference in presence of enlarged LNs between recurrence and non-recurrence group.

The method of treatment of patients with primary intussusception strikingly influences the recurrence rate. Surgical reduction is complicated by a recurrence rate of 1–3%, while the rate following hydrostatic reduction is 20% [16]. In our study, though the follow-up period was shorter, none of the cases who underwent operative reduction had recurrence. All the recurrent cases occurred following successful hydro-reduction. Thus, removal of an etiologic lesion if present, short period of postoperative adynamic ileus and postoperative adhesions might explain infrequency of recurrences after surgical reduction.

Nine cases recurred within first 24 h following successful hydro-reduction while four cases recurred within a month after reduction (Table 4). It is tempting to ascribe these early recurrences to incomplete reduction. However, we followed specific protocol for success of hydro-reduction and were convinced that all were completely reduced. Of the 13 recurrences, 9 patients were managed successfully with repeat hydro-reduction procedure and only 4 required operative reduction. The principal treatment of recurrent intussusception, in general, was the same as that of primary intussusception.

**Table 4:** Analysis of recurrence of intussusception.

	Total (N = 13)
Time of recurrence	
• 0–6 h	7
• 6–24 h	2
• 24 h- 1 month Etiology	4
Etiology	
• Idiopathic	12
• Meckel's diverticulum	1
Primary method of reduction	
• Hydro-reduction	13
• Operation	0
Treatment of recurrence	
• Hydro-reduction	9
• Operation	4

## 5 Limitations

There are several limitations to our study. First, the sample size was small because of low risk of recurrence. Second, there were some possibly unknown risk factors that we were unable to measure. The fact that pathological lead points are difficult to identify by abdominal sonography made us unable to evaluate pathological lead point as independent predictor of recurrence. Third, a short period of follow-up of recurrence was taken. Despite, these limitations, our study improves the understanding of risk factors for recurrence of intussusception in pediatric patients.

## 6 Conclusion

Children presenting with prolonged duration of symptoms more than 48 h, fever and palpable mass have higher likelihood of recurrence of intussusception. Parents of such children should be made aware of this possibility to recognize symptoms including urgent clinical attention by pediatric surgeons can avoid delayed presentations to hospital and help in better outcomes in pediatric patients.

## Ethical approval

Study was approved by the ethics committees of the Institutional review board of Tribhuvan University, Institute of Medicine.

## Sources of funding for your research

The authors declare that this study had no funding source.

## Author contribution

SA, DPK, RPP: Initiated the research, wrote the research proposal, conducted the research, did data entry and analysis, and wrote the manuscript. SA, DPK: Involved in the write-up of the methodology of the proposal and research work. SA: Contributed in analysis of data. SK, DPK, SN: Wrote and edited the manuscript. GD, RPP, DPK: Reviewed the manuscript. The authors read and approved the final manuscript.

## Consent

Written informed consent was obtained from the patient's guardians for publication of this study and accompanying images.

## Registration of research studies

1. Name of the registry: ClinicalTrails.gov.

2. Unique Identifying number or registration ID: NCT05259670.

3. Hyperlink to your specific registration (must be publicly accessible and will be checked): https://register.clinicaltrials.gov/prs/app/action/SelectProtocol?sid=S000BUVV&selectaction=Edit&uid=U000650N&ts=2&cx=-2djpx2.

## Guarantor

Dr. Dinesh Prasad Koirala.

## Provenance and peer review

Not commissioned, externally peer reviewed.

## Declaration of competing interest

No potential conflict of interest relevant to this article was reported.
